# The role of mutations in core protein of hepatitis B virus in liver fibrosis

**DOI:** 10.1186/1743-422X-6-209

**Published:** 2009-11-26

**Authors:** Ashraf Mohamadkhani, Ferdous Rastgar Jazii, Hossein Poustchi, Omidreza Nouraein, Shahsanam Abbasi, Masoud Sotoudeh, Ghodratollah Montazeri

**Affiliations:** 1 National Institute of Genetic Engineering and Biotechnology Tehran, Iran; 2 Digestive Disease Research Centre, Shariati Hospital, Medical Science/University of Tehran, Tehran, Iran

## Abstract

The core protein of hepatitis B virus encompasses B- and T-cell immunodominant epitopes and subdivided into two domains: the N-terminal and the functional C-terminal consisted phosphorylation sites. Mutations of the core gene may change the conformation of the core protein or cause alteration of important epitopes in the host immune response. In this study twenty nine men (mean age 40 ± 9 years old) with chronic hepatitis B were recruited for direct sequencing of the core gene. Serum ALT and HBV DNA level were measured at the time of liver biopsy. The effects of core protein mutations on patients' characteristics and subsequently mutations in B cell, T helper and cytotoxic T lymphocyte (CTL) epitopes and also C-terminal domain of core protein on the activity of liver disease was evaluated. Liver fibrosis was significantly increased in patients with core protein mutation (1.0 ± 0.8 vs 1.9 ± 1.4 for mean stage of fibrosis P = 0.05). Mutations in CTL epitopes and in phosphorylation sites of C-terminal domain of core protein also were associated with higher liver fibrosis (P = 0.003 and P = 0.04; Fisher's exact test for both). Patients with mutation in C-terminal domain had higher serum ALT (62 ± 17 vs 36 ± 12 IU/l, p = 0.02). Patients with mutations in B cell and T helper epitopes did not show significant difference in the clinical features. Our data suggests that core protein mutations in CTL epitopes and C-terminal domain accompanied with higher stage of liver fibrosis may be due to alterations in the function of core protein.

## Introduction

Worldwide, the 350 million people with chronic hepatitis B have a 15-25% risk of dying from HBV-related liver diseases, including cirrhosis and hepatocellular carcinoma [1]. It is evident that 70-84% of cirrhotic patients and 72% of individuals with hepatocellular carcinoma in Iran have evidence of exposure to HBV [2].

Naturally occurring mutations of hepatitis B virus (HBV) genome have an important role in the activity of HBV related liver diseases. Patients with long standing active liver disease are at high risk to develop liver cirrhosis or hepatocellular carcinoma [3]. The genome of hepatitis B virus encodes four overlapping open reading frames that are translated to viral core protein or HBc particle, the surface proteins, a reverse transcriptase (RT), and HBx [4]. The core protein is the major polypeptide of the nucleocapsid that during virus assembly polymerizes around a complex consisting of pregenomic mRNA and viral polymerase [5]. Core protein with genotype D which is frequent in Iran [6] holding 183 amino acids with a set of closely linked α-helices [7] and consists of two distinct domains, an N-terminal domain with 144 residues required for the assembly of the 32 nm nucleocapsid and a functional C-terminal domain [5,8]. Empty core shells made from truncated HBc at residue 149 revealed the important role of C-terminal in viral genome binding and nuclear transport of the core protein [9-11]. The C-terminal arginine-rich domain with a high similarity to protamin, consists of three repeated SPRRR motifs corresponded to the part of core protein that interact closely with RNA [5]. In this domain phosphorylated site residues located in amino acid sequences 155-183. Immature nucleocapsids which contain RNA are phosphorylated at six sites, while the mature nucleocapsids which contain DNA are completely dephosphorylated either inside cells or in extracellular virions [9]. This phosphorylation clearly plays an important role in the regulation of the function of C-terminal core protein [10,12]. Regard to HBc particles include into the HBV vaccines it can be demonstrated that core protein is a major target for antiviral immune response [13]. There is evidence that the HBc represents an important target for immune mediated viral clearance [14] by inducing B cell, T helper cell and cytotoxic T lymphocyte (CTL) responses [15]. Important recognition sites of the core protein are represented by the amino acid sequences 18-27, 88-96 and 141-151 for the CTL epitopes and amino acid sequences 1-20, 28-47, 50-69, 72-105 and 108-165 for T helper epitopes [15-17]. The immunodominant B cell recognition sites within HBcAg have been found around residues 74-89 and 126-135 [15,16]. Mutations in both B- and T-cell epitopes associated with viral persistence[16,18] influencing the host immune response and also the natural course of infection [19]. Previous studies showed that mutations in the region of the CTL epitopes promoted the immune selection pressure accompanied with particular clinical manifestations [20,21]. The Inflammatory activity produced by viral adaptive mechanism may persist in up to 15% of cases, leading to the development of cirrhosis [22].

Mutations in the functional C-terminal domain of the core protein might impact on other biochemical properties of this protein that have not been studied well. Considering the importance of the C-terminal core protein during viral replication that might be in interaction with some cellular proteins, our study objectives were to provide a pilot data in a group of male patients with chronic hepatitis B for the presence of mutations in the C-terminal of the core protein as well as in B cell, T helper and CTL epitopes of HBV core gene sequence and the effects of these mutations on the clinical, biochemical and virological parameters of patients. We also employed a computational prediction approach to define the function of the core protein.

## Materials and methods

### Human Subjects and Clinical Assessment

Chronic hepatitis B patients with HBeAg negative attending the Hepatitis Clinic of Shariati Hospital were evaluated. Twenty nine male subjects with detectable HBV DNA and candidate for liver biopsy were enrolled for the analysis of the prevalence of HBc mutations. The assessed laboratory parameters were included serum alanine aminotransferase (ALT) and viral load measured by standard methods. Serological markers for HBsAg, HBeAg were tested using commercially available enzyme-linked immunosorbent assay kits from RADIM (Italy). Liver biopsies from all patients were performed to define the stage of fibrosis using the modified HAI scoring system [23]. Serum samples were collected at the initial assessment before liver biopsy. Concurrence of hepatitis C virus and human immunodeficiency virus infections and autoimmune liver disease was excluded for all enrolled individuals. None of the patients received antiviral treatment prior to liver biopsy. Study protocol was approved by the Ethics Committee of our unit.

### Quantitative HBV DNA Assay and Direct Sequencing of Core Protein

HBV DNA was extracted from 200 μl of serum using QIAamp DNA Blood Mini Kit (QIAGEN USA). HBV DNA was then quantified in the Light-Cycler (Roche) using the RealART™ HBV LC PCR (QIAGEN, Hilden, Germany) according to the manufacturer's instructions. To amplify the nucleotide sequence encoding HBcAg, a pair of primers designed for PCR, the forward primer (position 374-392): 5'-TAGGAGGCTGTAGGCATAA-3' and the reverse primer (Position 1095-1114): 5'-GAACAGTAGAAGAATAAAGC-3'. Sequences were obtained by direct sequencing of a fresh PCR product on an ABI automated sequencer following concentration using a QIAEX II protocol (Qiagen, Crawley, UK).

### Analysis of Core Protein by Bioinformatics Tools

Deduced amino acid sequences of 29 core genes were aligned using CLUSTALX developed by the National Center for Biotechnology Information (NCBI, Bethesda, MD). Sequence similarity was assessed using BLASTP (NCBI, Bethesda, MD) with searching protein sequence databases. The nature of the kinase to interact with core protein was predicted by NetPhosK as a kinase-specific phosphorylation site predictor (online at http://www.cbs.dtu.dk/services/NetPhosK/). The biological function process of core protein was predicted by Protein Function Prediction (PFP) Version 2.0 beta release (online at http://dragon.bio.purdue.edu/pfp).

### Statistical Analysis

Continuous variables were compared using an independent t-test and categorical variables were compared using Fisher's exact test. The data are expressed as the mean ± SD. SPSS for Windows Version 14 (SPSS Inc, Chicago, USA) was used for all analyses. Two-tailed *P *value of < 0.05 was considered to be statistically significant.

## Results

### Clinical, Laboratory, and Virological Data of the Patients

The study group included 29 male subjects with a mean of 42 ± 9 years old. All patients had liver biopsies with the average length of 1.6 ± 0.8 cm and portal triads numbered 9 ± 6 per biopsy. HAI score and stage of fibrosis had a mean of 5.7 ± 2.4 and 1.6 ± 1.3 respectively (Table 1). Deduced amino acid sequences encoding the core protein of HBV from 29 patients against HBV genotype D consensus sequence showed that all patients were infected with genotype D. Nineteen of 29 (65.5%) patients had amino acid mutations in the full length of core protein while mutations in the phosphorylation site of the C-terminal were detected in 5 (17.5%). Amino acid residues 77, 80, 130 and 135 corresponded to B cell epitopes, amino acid residues 12, 35, 38, 64, 66, 113 and 116 to T helper epitopes and amino acid residues 93, 147 and 151 restricted to CTL epitopes [15,20]. Positions of mutations in deduced amino acid residues compared to consensus residue of genotype D in 29 patients are shown in Table 2.

**Table 1:** Analysis of clinical factors in relation to the presence of mutations in the core protein in 29 patients with chronic hepatitis B virus infection

Clinical factor	All patients *(n = 29)*	Wild Type *(n = 10)*	Mutant *(n = 19)*	*P value*
Age* (Years)	42 ± 9	43 ± 8	41 ± 9	*0.5*

ALT* (IU/l)	41 ± 16	34 ± 13	44 ± 16	*0.09*

HBV DNA* (Log copies/ml)	4.2 ± 0.8	4.0 ± 0.9	4.3 ± 0.8	*0.4*

HAI score*	5.7 ± 2.4	5.1 ± 2.2	6.1 ± 2.4	*0.2*

Stage of Fibrosis*	1.6 ± 1.3	1.0 ± 0.8	1.9 ± 1.4	*0.05*

*Mean ± SD

**Table 2:** Amino Acid mutation of HBc sequence deviated from HBV core gene.

	T helper epitopes						CTL epitopes				B cell epitopes				C-terminal	
**subject**	**T12**	**S35**	**Y38**	**E64**	**M66**	**E113**	**I116**	**M93**	**T147**	**R151**	**E77**	**A80**	**P130**	**P135**	**S176**	**S181**

**1**	-	-	-	-	-	-	-	-	-	-	-	-	-	-	-	-

**2**	-	-	-	D	I	-	-	-	-	-	-	-	-	-	-	P

**3**	-	-	-	-	-	-	-	-	-	-	-	-	-	-	-	-

**4**	-	-	F	-	-	-	-	-	-	-	Q	-	-	-	-	-

**5**	S	-	-	-	-	-	-	-	-	-	-	-	-	V	A	-

**6**	-	-	-	-	-	-	-	-	-	-	-	T	-	-	-	-

**7**	-	-	-	-	-	-	-	-	-	-	-	-	-	-	-	-

**8**	-	-	-	-	-	-	-	V	-	-	-	V	-	-	-	-

**9**	-	-	-	D	-	-	-	-	-	-	-	-	-	-	-	P

**10**	-	-	-	-	-	-	-	-	-	-	-	-	-	-	-	-

**11**	-	-	-	-	-	-	-	T	-	-	-	V	-	-	-	-

**12**	-	T	-	-	-	-	-	-	-	-	-	-	-	-	-	-

**13**	-	-	-	-	-	-	-	-	-	-	-	-	-	-	-	-

**14**	-	-	-	-	-	-	-	-	-	-	-	-	-	-	-	-

**15**	-	-	-	-	-	-	-	-	-	-	-	-	-	-	-	-

**16**	-	-	-	-	-	-	-	-	-	-	-	-	Q	-	-	-

**17**	-	-	-	D	-	-	-	-	-	-	-	-	-	-	-	-

**18**	-	-	-	-	-	-	-	-	-	-	-	-	-	-	-	-

**19**	-	-	F	-	-	-	-	-	-	-	-	-	-	-	-	P

**20**	-	-	-	-	-	-	L	-	-	-	-	-	-	-	-	-

**21**	-	-	-	-	-	-	-	-	-	-	-	-	-	-	-	-

**22**	-	-	-	D	-	-	-	-	-	-	-	-	-	Q	-	-

**23**	S	-	-	-	I	-	-	-	-	-	-	-	Q	-	-	-

**24**	-	-	-	-	-	-	-	-	-	-	-	-	-	-	-	-

**25**	-	-	F	-	-	-	-	-	-	R	-	-	-	-	-	P

**26**	-	-	-	-	-	Q	-	-	-	-	-	-	-	-	-	-

**27**	-	-	-	-	-	-	-	T	-	-	-	V	-	-	-	-

**28**	-	T	-	D	-	-	-	-	-	-	-	-	-	-	-	-

**29**	-	-	-	-	-	-	-	-	C	-	-	-	-	-	-	-

**Frequency (%)**	2(6.9)	2(6.9)	3(10.3)	5(17.2)	2(6.9)	1(3.4)	1(3.4)	3(10.3)	1(3.4)	1(3.4)	1(3.4)	4(13.8)	2(6.9)	2(6.9)	1(3.4)	4(13.8)

The consensus sequence of genotype D is shown in the first line. Dashes represent residues identical to the reference residues.

### Association of Core Protein Mutations with the Outcome of HBV Infection

Comparisons of various clinical features in terms of the presence of mutations in the core protein are presented in Table 1. The presence of mutations in core protein was associated with higher serum ALT although this was not significant. However liver fibrosis significantly increase in patients with core protein mutation (1.0 ± 0.8 vs 1.9 ± 1.4 P = 0.05). When the Immunodominant epitopes and C-terminal domain of HBV core protein were separately analyzed the mutation of CTL epitopes showed higher viral replication (4.1 ± 0.8 vs 4.9 ± 0.7 log copies/ml, p = 0.05). There was no significant difference in the clinical features of patients with mutation in B cell and T helper epitopes. We observed a significantly increased serum ALT of patients with mutations in C-terminal domain (36 ± 12 vs 62 ± 17 IU/I, P = 0.02). Table 3 presented the clinical findings of chronic hepatitis B patients in correlation with mutations in Immunodominant epitopes and C-terminal domain of HBV core protein. The mean of HAI score and fibrosis stage in subjects with mutations in C-terminal domain when compared with those without this mutations were 7.6 ± 2.2 vs 5.3 ± 2.2, p = 0.06 and 3.4 ± 1.1 vs 1.3 ± 0.7, p = 0.02. However, we found no significant difference between the mean score of HAI and fibrosis stage with mutations in CTL epitopes.

**Table 3:** The correlation of mutations in Immunodominant epitopes and C-terminal domain of HBV core protein with clinical finding of 29 chronic hepatitis B patients

	Immunodominant epitopes Wild type/Mutant**						Wild type/Mutant**	
**Mutation site**	**B cell**	P*	**CTL**	P*	**T helper**	P*	**C-terminal domain**	P*

**Frequency (%)**	20/9 (68/32)		24/5 (82/17)		16/13 (55/45)		24/5 (82/17)	

**Age (Years)**	42 ± 8/40 ± 9.6	*0.5*	42 ± 8.3/38 ± 9.3	*0.3*	42 ± 8.7/41 ± 8.5	*0.5*	41 ± 8/44 ± 10	*0.6*

**ALT (IU/l)**	42 ± 18/38 ± 11	*0.4*	39 ± 14/48 ± 21	*0.4*	35 ± 13/47 ± 17	*0.06*	36 ± 12/62 ± 17	***0.02***

**HBV DNA** **(Log copies/ml)**	4.2 ± 0.7/4.2 ± 1	*0.9*	4.1 ± 0.8/4.9 ± 0.7	***0.05***	4.3 ± 0.9/4.2 ± 0.7	*0.6*	4.2 ± 0.9/4.5 ± 0.3	*0.2*

**HAI score**	6 ± 2.4/5.2 ± 2.4	*0.4*	5.4 ± 2.3/7.4 ± 2.1	*0.1*	5.6 ± 2/5.8 ± 2.8	*0.6*	5.3 ± 2.2/7.6 ± 2.2	*0.06*

**Stage of Fibrosis**	1.7 ± 1.3/1.4 ± 1	*0.4*	1.3 ± 1/2.8 ± 1.4	*0.3*	1.3 ± 0.9/1.9 ± 1.5	*0.2*	1.3 ± 0.7/3.4 ± 1.1	***0.02***

*t Test. **Mean ± SD

To examine the relationship of these mutations with the stage of fibrosis, patients were classified based on the score of liver fibrosis less and more than 2 (set as cut off point). As illustrated in Table 4, statistical analysis of the relationships between the mutations in the core gene and the stage of fibrosis showed that mutations in C-terminal domain with codon 181 being most frequently affected were significantly associated with development of liver fibrosis. Mutations in CTL epitopes of core protein also associated with higher liver fibrosis (P = 0.003 and P = 0.04; Fisher's exact test for both).

**Table 4:** The relationships between the mutations in the core gene and the Stage of fibrosis

Core region	Stage of Fibrosis	Patients with mutation in core protein (n = 19)		
***Mutations in:***	***Mean ± SD***	***Fibrosis score <2(%)***	***Fibrosis score >2 (%)***	P-value*

**Core protein**	1.9 ± 1.3	13(68)	6(32)	0.06

**T helper epitopes**	1.9 ± 1.5	9(69)	4(31)	0.3

**CTL epitopes**	2.8 ± 1.4	2(40)	3(60)	**0.04**

**B cell epitopes**	1.4 ± 1.1	7(77)	2(23)	1.0

**C-terminal domain**	3.4 ± 1.1	1(20)	4(80)	**0.003**

*Fisher's exact test P-value

^#^Number (%)

### Functional Analysis of Core Protein

**Figure 1:**
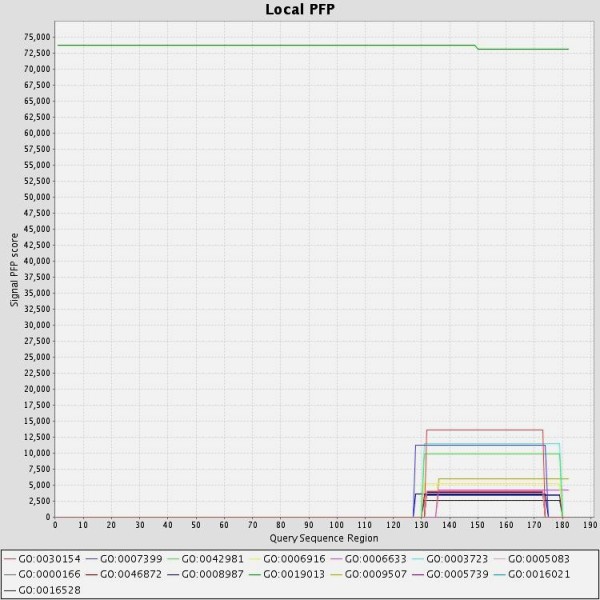
**Predicted GO annotations for HBV core protein sequence, The PFP algorithm scored GO terms individually and includes information from distantly related sequences to HBV core protein**. The function of each GO has been shown in table 5.

Core protein with the largest number of serine sites in C-terminal tail could be widely phosphorylated by kinases. Amino acid residues 176 and 181 which were defined as mutation sites in current study had high score performance value for different kinases presented by NetPhosK predictor. PFP algorithm searched conventional databases with relative probability of Gene Ontologies (GO) to predict the most probable GO annotations in three Biological Process (BP), Molecular Function (MF) and Cellular Component (CC) categories which is presented in Fig. 1 and Table 5. According to this prediction the feature of viral nucleocapsid with the highest score of 73709 in CC category related to the full length of core protein while the function of core protein in two other categories BP and MF limited to C-terminal domain.

**Table 5:** Prediction scores for top 5 predictions function of HBV core protein in each GO category

GO term	Short Def	Raw Score	Term Type
**GO0030154**	cell differentiation	13589.15	BP

**GO0007399**	neurogenesis	11229.85	BP

**GO0042981**	regulation of apoptosis	9909.73	BP

**GO0006916**	anti-apoptosis	5241.9	BP

**GO0006633**	fatty acid biosynthesis	4198.2	BP

**GO0003723**	RNA binding	11434.09	MF

**GO0005083**	small GTPase regulatory/interacting protein activity	4155.72	MF

**GO0000166**	nucleotide binding	3985.78	MF

**GO0046872**	metal ion binding	3868.07	MF

**GO0008987**	quinolinate synthetase A activity	3672.6	MF

**GO0019013**	viral nucleocapsid	73709.99	CC

**GO0009507**	chloroplast	6016.97	CC

**GO0005739**	mitochondrion	3530.83	CC

**GO0016021**	integral to membrane	3426.14	CC

**GO0016528**	sarcoplasm	2591.69	CC

## Discussion

Mutant HBV could display enhanced virulence with increased levels of HBV replication, or alteration of epitopes which is important in the host immune response. Sequence variation in core protein is one of the powerful viral strategies for escaping recognition by the host's immune response linked to virus persistence or severity of chronic hepatitis B infection [18]. The reason for selection of amino acid changes in hepatitis B virus proteins, as well as their functional or immunological relevance is speculative [24]. Through the evolution, most functional DNA is expected to have attained a sequence that is near optimal for its environment [25]. Previous work from Iran has indicated a high nucleotide identity for HBV isolates from 98.4 to 100% [6] in overall that suggests mutations in the HBV core gene sequence were more likely resulted from natural selection during the course of infection.

We detected mutations in immunedominant epitopes and C-terminal domain of core protein in 65.5% of our patients with chronic hepatitis B. The results revealed increased liver fibrosis in patients with mutation in both C-terminal domain and CTL epitopes. The mean of fibrotic stage found to be highest in patients with mutations in phosphorylation sites of C-terminal domain. Patients with mutations in the CTL epitopes accompanied with active viral replication. A proposed mechanism for this observation is that selection of mutations in the CTL epitopes alters core antigenicity that results in not to be recognized by the corresponding immune response and consequently induce a new immune response as evidenced by the high levels of HBV DNA recorded in such cases. Patients with mutation in C-terminal domain of core protein had higher level of ALT than their counterparts. Elevated aminotransferase values reflect increased histologic necroinflammatory disease activity.

The structure of core protein is largely α-helical rods with C-terminal basic tail that interact closely with viral RNA pregenome or the viral DNA genome [11]. The C-terminal of the core protein is highly conserved with repetitive structure that is required for many aspects of viral production. This may be due to its functional significance, as well as to the overlapping P gene [8,10]. HBV core protein appears as a phosphoprotein and many kinases have been reported to be associated with viral capsid [26]. This was confirmed by NetPhosK predictor tool in which C-terminal of core protein is phosphorylated by multiple protein kinases to interact with intracellular protein. Phosphorylation is the most important and best understood modification to modulate protein activity and signal propagation for homeostasis processes like cell cycle progression, differentiation, development and peptide hormone response [27]. The maturation stage of the HBV has been shown to be correlated to the phosphorylation state of core molecules [12]. This phosphorylation clearly plays an important role in the regulation of C-terminal domain function [28].

To explain the effect of mutations of core protein in the biological process of the cell, we predicted the major functions of the core protein of hepatitis B virus. Protein prediction function showed that viral nucleocapsid activity has the highest score for the whole of HBc molecule in cellular component GO category (Fig. 1). This is consistent with experimental data that the HBc sequence 1-144 was sufficient for self assembly and HBV pregenome encapsidation but not for binding to the viral pregenome or the viral DNA genome and the production of relaxed circular HBV DNA [5,10].

Furthermore, other predicted functions of HBc were limited to C-terminal domain which is rich in phosphorylation sites (Fig. 1). We suggested that the molecular function and biological process of core protein are more affected by mutations mainly in phosphrylation sites of C-terminal domain. Usuda et al showed that HBV core proteins (p21c) from symptom-free carriers have a higher extent of phosphorylation than hepatitis patients [29] which is consistent with our finding in this study. In this study, 4 out of 5 mutations in the phosphorylation sites of the C-terminal core protein related to a proline replacing a serine residue at position 181 that is particularly frequent in HBV of patients with hepatocellular carcinoma or end stage liver disease [30,31]. It was demonstrated by Kim et al, that the intracellular level of HBx can be downregulated by HBc via a novel mechanism involving the activation of the proteasome-mediated degradation of HBx. They showed that the C-terminal half of HBc is responsible for its inhibitory effect and suggested that HBc act as a novel regulator of the HBV life cycle and hepatocellular carcinogenesis [32]. Mutation of phosphorylation sites in the C-terminal domain might change the regulatory effects of HBc on HBx and consequensing HBx-mediated apoptosis that could be interpreted by predicted apoptotic regulatory function of HBc. Some unknown secondary structure as a result of mutation in the core protein may determine its role in the biological function and replication of hepadnavirus. Additional in vivo studies for the effects of core protein mutations on cellular function are required to determine its precise impact on the development of liver fibrosis. These findings may provide useful insights for the design of improved drug for the treatment of HBV infection.

## Conclusion

In conclusion, HBV populations with combinations of mutations in phosphorylation site in C-terminal domain and CTL epitopes of core protein were associated with more severe liver fibrosis.

## Abbreviations used in this paper

(HBV): Hepatitis B virus; (CHB): Chronic hepatitis B; (CTL): Cytotoxic T lymphocyte.

## Competing interests

The authors declare that they have no competing interests.

## Authors' contributions

AM was responsible for research design, sequence analysis, interpretation, and writing of this manuscript, FR was the principal investigator and is primarily responsible for all aspects of the funding, HP coordinated sample collection, SA and ON carried out PCR and ELISA based assays, liver biopsy specimens reviewed by MS and GM contributed with critical reading. All authors read and approved the final version.
